# Simplex cerebral cavernous malformations with *MAP3K3* mutation have distinct clinical characteristics

**DOI:** 10.3389/fneur.2022.946324

**Published:** 2022-08-26

**Authors:** Ran Huo, Jie Wang, Ying-Fan Sun, Jian-Cong Weng, Hao Li, Yu-Ming Jiao, Hong-Yuan Xu, Jun-Ze Zhang, Shao-Zhi Zhao, Qi-Heng He, Shuo Wang, Ji-Zong Zhao, Yong Cao

**Affiliations:** ^1^Department of Neurosurgery, Beijing Tiantan Hospital, Capital Medical University, Beijing, China; ^2^China National Clinical Research Center for Neurological Diseases, Beijing, China

**Keywords:** cerebral cavernous malformations, *MAP3K3* mutation, CCM gene mutations, clinical characteristics, thrombomodulin, tight junctions

## Abstract

**Objectives:**

To investigate the clinical characteristics of cerebral cavernous malformations (CCMs) with *MAP3K3* somatic mutation.

**Methods:**

We performed a retrospective review of our CCMs database between May 2017 and December 2019. The patients with simplex CCMs identified to harbor a *MAP3K3* or CCM gene somatic mutation were included. Clinical characteristics were recorded. Univariate and multivariate logistic analyses were used to assess the risk factors associated with hemorrhage events of CCMs. To explore the underlying mechanism, we transfected MEKK3-I441M-overexpressing and *CCM2*-knockdown lentiviruses into human umbilical vein endothelial cells (HUVECs) and investigated thrombomodulin (TM) and tight junctions (TJs) protein expression by western blotting and immunofluorescence. Finally, immunohistochemistry was used to validate TM and TJs protein expression in surgical samples.

**Results:**

Fifty simplex CCMs patients were included, comprising 38 *MAP3K3* mutations and 12 CCM gene mutations. Nine (23.7%) patients with *MAP3K3* mutations and 11(91.7%) patients with CCM gene mutations exhibited overt hemorrhage, respectively. Multivariate logistic analyses revealed that *MAP3K3* mutation was associated with a lower risk of hemorrhage events. In the *vitro* experiments, ZO-1 expression was not reduced in MEKK3-I441M-overexpressing HUVECs compared with wild type, whereas it was significantly decreased in *CCM2*-knockdown HUVECs compared with control. In the MEKK3-I441M-overexpressing HUVECs, TM expression was increased, and the NF-κB pathway was significantly activated. After treatment with an NF-κB signaling inhibitor, TM expression was further upregulated. Meanwhile, TM expression was increased, but the NF-κB pathway was not activated in *CCM2-*knockdown HUVECs. Accordingly, immunohistochemistry showed that ZO-1 expression in the *MAP3K3-*mutant samples was significantly higher than that in the CCM-mutant samples. TM expression in the *MAP3K3*-mutant lesions was significantly lower than that in the CCM-mutant samples.

**Conclusion:**

Simplex CCMs with *MAP3K3* mutation occasionally present with overt hemorrhage, which is associated with the biological function of *MAP3K3* mutation.

## Introduction

Cerebral cavernous malformations (CCMs) of the central nervous system are vascular anomalies affecting 0.16 to 0.5% of the general population (1, 2). These lesions show cerebral venous capillary dysplasia with endothelial clusters filled with blood and susceptible to hemorrhage (3, 4). Several studies have reported that the hemorrhage rates of CCMs vary from 1.6 to 4.5% per patient-year (3, 5, 6). Because of lesion bleeding, CCM lesions frequently lead to epileptic seizures, headaches, focal neurological deficits, or life-threatening strokes (7, 8).

Approximately 20% of CCMs are inherited in an autosomal dominant manner (familial CCMs), while 80% of CCMs occur without a family history (simplex CCMs) (9–11). Somatic mutations in CCM genes (*CCM1/KRIT1, CCM2/MGC4607*, and *CCM3/PDCD10*) were reported early in simplex CCM lesions (12), and recent studies reported that over one-third of simplex CCMs contain the *MAP3K3* (c.1323C>G [p. Ile441Met]) somatic mutation (13, 14); the *MAP3K3* and CCM gene mutations were mutually exclusive (13). CCMs arise from the endothelial gain of MEKK3-KLF2/4 signaling, while *MAP3K3* and CCM gene mutations both activate the MEKK3-KLF2/4 signaling pathway (13, 15, 16). Whether *MAP3K3* mutation has distinct features compared with CCM gene mutations must be investigated.

In this study, we retrospectively analyzed the clinical data of 50 patients with simplex CCMs identified to harbor a CCM gene or *MAP3K3* somatic mutation. Meanwhile, we investigated the expression of thrombomodulin (TM) and tight junctions (TJs) proteins in HUVECs in the *vitro* experiments and surgical samples between *MAP3K3* and CCM gene mutations. Our study indicates that, compared with CCM gene mutations, *MAP3K3* mutant simplex CCMs have different clinical characteristics.

## Methods

### Study design and patients

We performed a retrospective review of our CCMs database between May 2017 and December 2019. This study was performed according to an institutional review board-approved protocol in compliance with local and institutional regulations for the study of human subjects. Written informed consent was obtained from all participating patients (or guardians of patients). The patients with simplex CCMs identified to harbor a *MAP3K3* or CCM gene mutation were included consecutively. Patients with available presurgical MRI with poor quality or a gamma knife radiosurgery history were excluded.

### Data collection

The demographic and clinical information of patients with simplex CCMs including age, sex, main complaint and lesion location, size, Zabramski type, concurrent with developmental venous anomaly, and overt hemorrhage was recorded and analyzed. Based on the 1994 Zabramski classification, all CCMs lesions were defined as Type I-IV (17). According to previous studies, a hemorrhage event was defined as a symptomatic event with radiographic evidence of overt intracerebral hemorrhage (5, 11).

### Culture and treatment of human umbilical vein endothelial cells (HUVECs)

Commercially available HUVECs (#8000; ScienCell) were cultured according to the manufacturer's guidelines and were not used beyond P8. HUVECs were cultured in an endothelial cell medium (ECM; #1001; ScienCell) and were maintained in a humidified atmosphere at 37°C in 5% CO_2_. Pyrrolidinedithiocarbamate ammonium, an inhibitor of nuclear factor NF-κB signaling (S3633; 100 μM; Selleck), and doramapimod, an inhibitor of p38 signaling (S1574; 500 nM; Selleck), were directly added to the endothelial cell medium.

### Lentivirus transfection

Our study reported that MAP3K3 mutation and CCM gene mutation in the CCMs lesions existed mutually exclusive (13), and therefore, the effect of *CCM2* knockdown and *MAP3K3* mutation was explored in HUVECs, respectively. MEKK3-I441M-overexpressing or *CCM2*-knockdown lentiviruses were obtained from SyngenTech (Beijing, China). HUVECs were plated in 6-well dishes at 50% confluence and were infected with MEKK3-I441M-overexpressing lentivirus (termed oeI441M), wild-type MEKK3 lentivirus (termed WT), *CCM2*-knockdown lentivirus (termed sh*CCM2*), or a negative control (termed shNC). Pools of stable transductions were generated by selection using puromycin (2 μg/ml) for 2 weeks.

### Western blotting

Proteins from cultured cells were extracted using radioimmunoprecipitation assay lysis buffer and were quantified using a bicinchoninic acid protein assay kit. Next, equal amounts of protein were separated by sodium dodecyl sulfate–polyacrylamide gel electrophoresis and transferred to a polyvinylidene fluoride transfer membrane. After blocking, the membranes were incubated with the following primary antibodies: *CCM2* (1:1000; 123930; Abcam), MEKK3 (1:1000; #5727; CST), TM (1:1000; 109189; Abcam), phospho-NF-κB (1:1000; #3033; CST), ZO-1 (1:750; #13663; CST), Occludin (1:1000; #91131; CST), Claudin-5 (1:1000; 131259; Abcam), VE-cadherin (1:1000; #2500; CST), phospho-p38 (1:1000; #4511; CST), phospho-ERK5 (1:1000; #3371; CST), KLF2 (1:500; 17008; Abcam), KLF4 (1:750; #12173; CST), and GAPDH (1:1000; #5174; CST). The membranes were then incubated with the appropriate secondary antibodies. The proteins were visualized using electrochemiluminescence (ECL) reagents. The blots were quantified by densitometry using ImageJ (NIH Image, Bethesda, MD).

### Immunofluorescence staining

For immunofluorescence staining, cell lines were grown on glass coverslips for 48 h and fixed with 4% paraformaldehyde. After permeabilization in 0.3% Triton X-100 and blocking, the cells were exposed to primary antibodies against ZO-1 (1:100; #13663; CST) and TM (1:200; #109189; Abcam) at 4°C overnight and goat anti-rabbit IgG-H&L (1:1,000; M21002; Abmart) at room temperature for 1 h. 4′,6-Diamidino-2-phenylindole was used to label cell nuclei. The slides were imaged using an inverted microscope (IX71; Olympus, Japan). ImageJ (NIH Image, Bethesda, MD) was used to quantitate the expression of proteins.

### Immunohistochemistry

Histological sections were obtained from our sample bank as described in our previous study (13, 18) and included 3 *MAP3K3* mutant surgical samples, 3 CCM gene mutant surgical samples, and 3 superficial temporal arteries as normal controls. The slides were incubated with primary antibodies against TM (1:200; #109189; Abcam), ZO-1 (1:500; #221547; Abcam), Claudin-5 (1:200; #131259; Abcam), VE-cadherin (1:200; #2500; CST), Endoglin (1:500; #252345; Abcam), VEGF (1:100; #0265; ZSGB-BIO), PCNA (1:100; #0213; ZSGB-BIO), HIF-alpha1 (1:300; #463; Abcam), and Flk1 (1:200; #10012-T24; Sino-Biological) overnight at 4°C and then were incubated with a biotinylated secondary antibody at room temperature for 1 h followed by horseradish peroxidase-labeled streptavidin for 30 min. After the sections were washed with Tris buffer, they were stained with 3,3′-diaminobenzidine, and the nuclei were counterstained with hematoxylin. Images were acquired using a Zeiss Axio Scope A1 microscope. Two authors (R. H. and J. W.) blinded to the mutation status analyzed the immunohistochemical staining for ZO-1, TM, Claudin-5, and VE-cadherin in the recruited patients. Three randomly selected fields in non-adjacent tissue sections per tissue specimen were analyzed as described previously (18, 19). A positive reaction was indicated by a brown color using 3,3′-diaminobenzidine. ImageJ (NIH Image, Bethesda, MD) was used to calculate the integrated optical density.

### Statistical analysis

Continuous variables were presented as medians and interquartile range (IQR) or as means ± SD, and categorical variables were expressed as percentages. The Wilcoxon rank-sum test, *t*-test, chi-squared (χ^2^) test, and Fisher's exact test were used accordingly. Univariate and multivariate logistic regression analyses were used to assess the risk factors for hemorrhage events of CCMs. Variables with *p* < 0.20 in the univariate analysis were then used in the multivariate analysis. Analyses were performed using the statistical software SPSS 24.0 (IBM Corp, Armonk, NY, USA) and PRISM (GraphPad, version 8.0). A 2-tailed *p* < 0.05 was considered statistically significant.

## Results

### *MAP3K3* mutation was associated with a lower risk of hemorrhage events compared with CCM gene mutation

**Figure 1:**
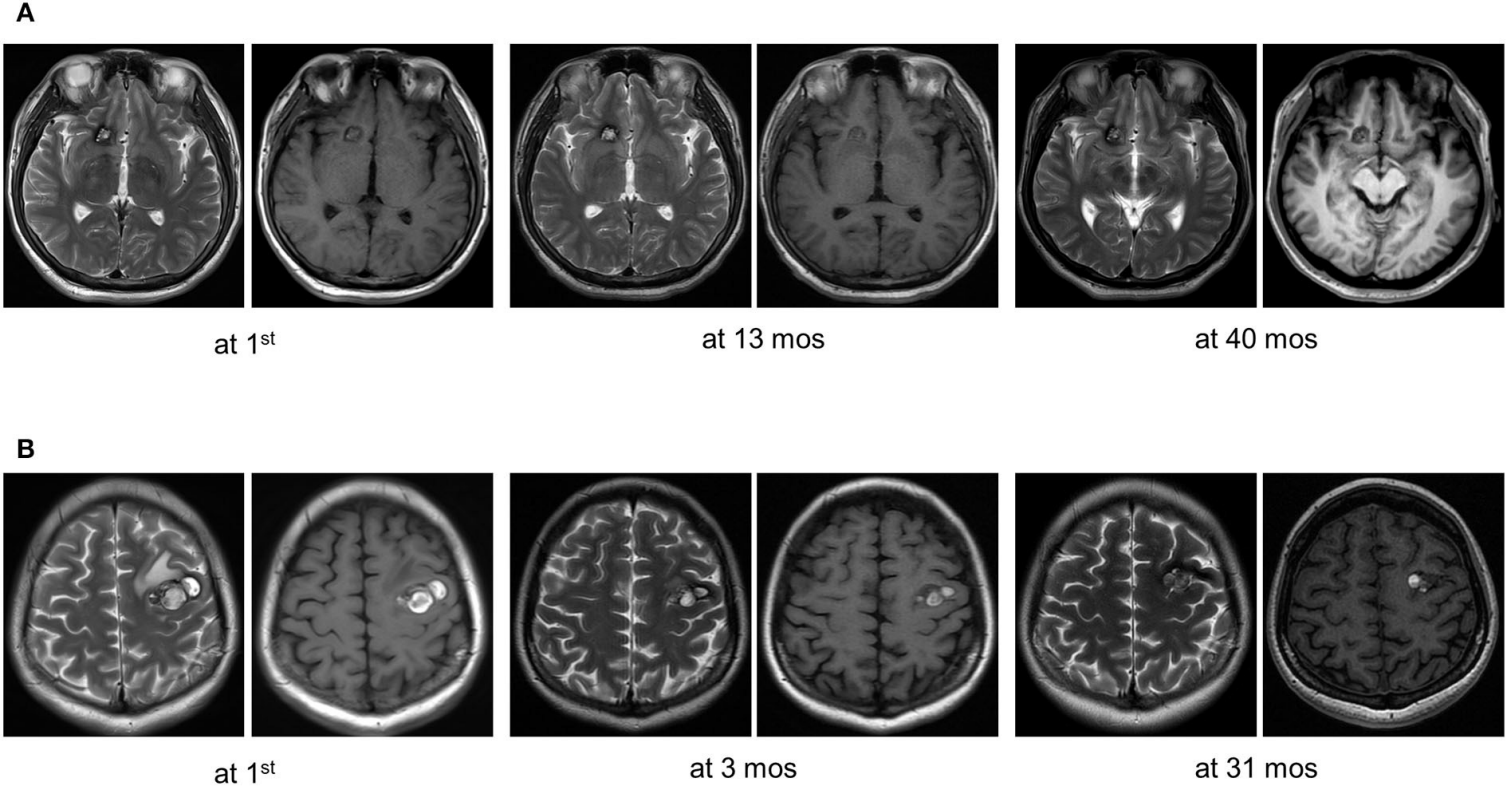
Representative follow-up MRI scans of *MAP3K3* mutation and CCM gene mutation lesions before surgical resection. **(A)** A patient who harbored a *MAP3K3* mutation lesion remained stable based on follow-up MRI scans at 13 and 40 months. **(B)** A patient who harbored a *CCM1* mutation lesion exhibited recurrent overt hemorrhage based on follow-up MRI scans at 3 and 31 months.

Fifty patients with simplex CCMs were included, comprising 38 (76.0%) recurrent *MAP3K3* somatic mutations and 12 (24.0%) CCM gene somatic mutations. Among the 50 patients, 25 (50%) patients had information about *PIK3CA* mutation, and 14 of the 25 patients harbored *PIK3CA* mutation. CCM gene mutation, *MAP3K3* mutation, and *PIK3CA* mutation with their mutant allele frequencies are shown in Supplementary Table 1. The clinical information of 50 patients was presented in Table 1. Nine (23.7%) of the 38 patients with *MAP3K3* somatic mutations presented with symptomatic events and corresponding overt intracerebral hemorrhage on the magnetic resonance imaging (MRI), whereas 11(91.7%) of the 12 patients with CCM gene somatic mutations exhibited overt intracerebral hemorrhage (13). Multivariate logistic regression analyses showed that compared with the CCM gene mutation, *MAP3K3* somatic mutation was associated with a lower risk of hemorrhage events [OR = 0.028 (95% CI: 0.003–0.255); *p* = 0.001; Table 2]. Among the 25 patients with information about *PIK3CA* mutation, *PIK3CA* mutation was not associated with hemorrhage events [OR = 0.581 (95% CI: 0.114–2.872); *p* = 0.497; Table 2]. Of the 50 patients, 15 of the 38 patients who harbored *MAP3K3* mutation and 5 of the 12 patients who harbored CCM gene mutation had repeated follow-up MRI scans before surgical resection (mean follow-up duration: 15.7 ± 24.4 months vs. 13.2 ± 15.8 months; *p* = 0.834). These data showed that *MAP3K3* mutation CCM lesions remained stable with Zabramski classification type II, while CCM gene mutation lesions presented with repeated overt intracerebral hemorrhage. The representative follow-up MRI scans are shown in Figure 1A (*MAP3K3* mutation lesion) and Figure 1B (CCM gene mutation lesion), respectively. These findings implied that *MAP3K3* mutations were associated with a lower risk of hemorrhage events, which was different from CCM gene mutation.

**Table 1:** Baseline characteristics of the 50 patients.

**Variables**	**Overall**	***MAP3K3* mutation**	**CCM gene mutation**	***p*-values**
	**(*n* = 50)**	**(*n* = 38)**	**(*n* = 12)**	
Age-mean-yr	33.4 ± 16.0	31.9 ± 16.3	38.4 ± 14.6	0.221^†^
Female-no. (%)	18 (36.0)	12 (31.6)	6 (50)	0.416^‡^
Location-no. (%)				0.036^§^^*^
Multiple CCMs	2 (4.0)	0	2 (16.7)	
Lobar	33 (66.0)	27 (71.1)	6 (50.0)	
Deep	4 (8.0)	2 (5.3)	2 (16.7)	
Cerebellum	2 (4.0)	1 (2.6)	1 (8.3)	
Brainstem	9 (18.0)	8 (21.1)	1 (8.3)	
DVA-no. (%)	12 (24.0)	8 (21.1)	4 (33.3)	0.631^‡^
Hemorrhage events-no. (%)	20 (40.0)	9 (23.7)	11 (91.7)	<0.001^‡^^*^
Size-mean-mm	23.7 ± 9.3	23.3 ± 9.6	25.0 ± 8.4	0.904^†^
Main complaint-no. (%)				0.873^§^
Epilepsy	25 (50.0)	20 (52.6)	5 (41.7)	
FND	14 (28.0)	10 (26.3)	4 (33.3)	
Headache	6 (12.0)	4 (10.5)	2 (16.7)	
Others	5 (10.0)	4 (10.5)	1 (8.3)	
Zabramski type-no. (%)				<0.001^§^^*^
Type I	17 (34.0)	7 (18.4)	10 (83.3)	
Type II	27 (54.0)	27 (71.1)	0	
Others	6 (12.0)	4 (10.5)	2 (16.7)	

CCMs, cerebral cavernous malformations; DVA, developmental venous anomaly; FND, focal neurological deficit; *MAP3K3* mutation, *MAP3K3* (c.1323C>G [p.Ile441Met]) somatic mutation; CCM gene mutation, *CCM1/KRIT1* or *CCM2/MGC4607* somatic mutation.

† t-test.

‡ chi-square test.

§ Fisher's exact test.

* p <0.05.

**Table 2:** Univariate and multivariate analyses of risk factors associated with hemorrhage events of CCMs.

	**Univariate analysis**		**Multivariate analysis**	
**Variable**	**OR (95%CI)**	***p*-value**	**OR (95%CI)**	***p*-value**
Age	1.000 (0.965–1.037)	0.983		
Female	1.333 (0.412–4.310)	0.631		
Size	1.011 (0.949–1.076)	0.739		
Location				
Lobar^®^				
Deep	2.000 (0.248–16.159)	0.516		
Cerebellum	2.000 (0.114–35.089)	0.635		
Brainstem	1.600 (0.357–7.177)	0.539		
DVA	2.692 (0.713–10.170)	0.144		
Genotype				
CCM gene mutation^®^				
*MAP3K3* mutation	0.028 (0.003–0.249)	0.001^*^	0.028 (0.003–0.255)	0.001^*^
*PIK3CA* mutation^‡^	0.581 (0.114–2.872)	0.497		

CCMs, cerebral cavernous malformations; DVA, developmental venous anomaly; ^®^, reference.

‡ The reference was lesions without *PIK3CA* mutation.

* p <0.05.

Additionally, we explored the differences observed in *MAP3K3* and CCM gene mutation related to clinicopathological features (hemorrhagic episodes, lesion size, and symptoms) between the brainstem and supratentorial CCM lesions. In our study, there were 9 lesions with CCM gene mutation located in the brainstem and supratentorial and 37 lesions with *MAP3K3* mutation located in the brainstem and supratentorial. In the CCM gene mutation group, no differences were observed in hemorrhagic episodes and symptoms between the brainstem and supratentorial CCM lesions; the difference in lesion size between the brainstem and supratentorial CCM was not available because of limited data. In the *MAP3K3* mutant group, patients with lesions located in the brainstem were more likely to undergo focal neurological deficit than supratentorial CCM lesions, and there was no difference observed in hemorrhagic episodes and lesion size between the brainstem and supratentorial CCM lesions (Supplementary Table 2).

### *MAP3K3* mutation has different effects on ZO-1 expression compared with *CCM2* knockdown in HUVECs

Recurrent hemorrhage was the major presentation feature of CCMs, and the mechanisms underlying the hemorrhage of CCMs include loss of cell-cell junctions and local increase in the antithrombotic molecule. CCM endothelium was associated with unstable endothelial cell-cell contacts and locally elevated expression of anticoagulant endothelial receptors TM (20–24). We hypothesized that *MAP3K3* mutations were associated with a lower risk of hemorrhage events that might result from the biofunction features of the *MAP3K3* mutation concerning the expression of TM and TJ proteins.

**Figure 2:**
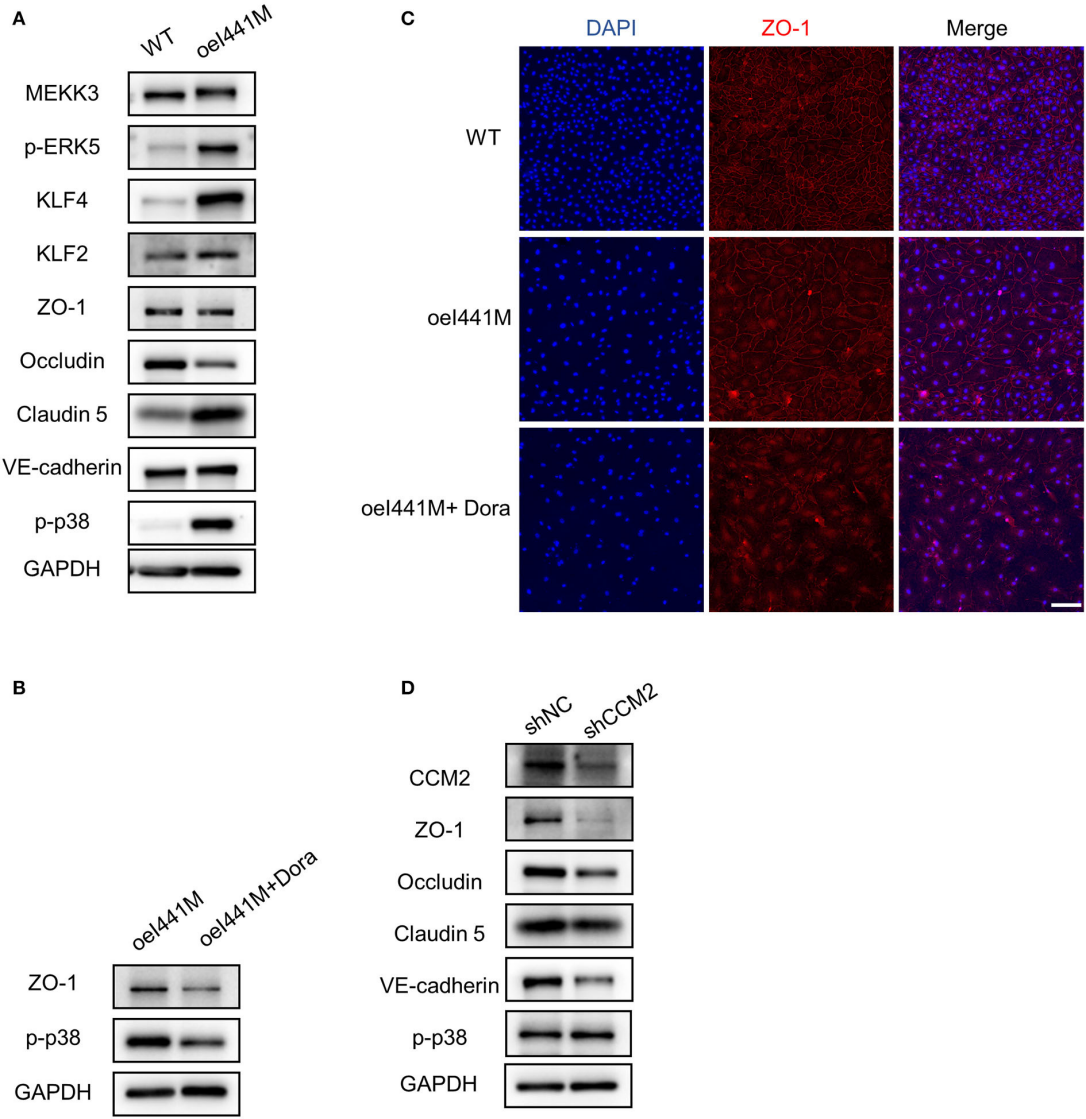
*MAP3K3* mutation has different effects on ZO-1 expression compared with *CCM2* knockdown in HUVECs. **(A–C)** HUVECs were infected by lentivirus with wild-type MEKK3 (WT) or MEKK3-I441M overexpression (oeI441M). Panel A shows that the expression of phospho-p38 (p-p38), phospho-ERK5 (p-ERK5), KLF2, and KLF4 was increased, but the expression of ZO-1, Claudin-5, and VE-cadherin was not decreased in MEKK3-I441M-overexpressing HUVECs, as shown by Western blotting. Panels B and C show that after treatment with doramapimod (Dora), an inhibitor of p38 signaling, the expression of ZO-1 was significantly downregulated. **(D)** Western blotting showed that compared with shNC, the expression of ZO-1, Claudin-5, Occludin, and VE-cadherin was significantly decreased in *CCM2*-knockdown HUVECs. HUVECs were infected with *CCM2*-knockdown lentivirus (sh*CCM2*) or a negative control (termed shNC). One representative experiment of 3 is shown. Scale bar, 200 μm.

To investigate the expression of cell-cell junction proteins in *MAP3K3* c.1323C>G mutation and *CCM2* knockdown endothelial cells, we infected HUVECs with lentivirus overexpressing MEKK3-I441M (*MAP3K3* encodes MEKK3), wild-type MEKK3 (WT), knockdown *CCM2* (sh*CCM2*), and negative control (shNC), respectively. Compared with the WT group, the expression of ZO-1, an essential cell-cell junction protein that plays a vital role in TJs formation, was not reduced after MEKK3-I441M overexpression, as shown by Western blotting and immunofluorescence staining (Figures 2A,C and Supplementary Figures 1A,C), and after MEKK3-I441M overexpression, Occludin expression was reduced, whereas Claudin-5 and VE-cadherin expression levels were not reduced (Figure 2A and Supplementary Figure 1A). Previous studies have indicated that high KLF2 and KLF4 expression levels may result in decreased ZO-1 expression and that p38 activation can increase ZO-1 expression (20, 25, 26). Our previous findings suggested that MEKK3-I441M enhances ERK5-KLF2/4 and p38 signaling, while *CCM2* knockdown only activated ERK5-KLF2/4 signaling (13). Therefore, we hypothesized that MEKK3-I441M could downregulate ZO-1 expression by increasing KLF2/4 expression while upregulating ZO-1 expression by activating p38 signaling. Western blotting showed that the KLF2, KLF4, and phospho-p38 levels were significantly increased after MEKK3-I441M overexpression compared with those in the wild-type group (Figure 2A and Supplementary Figure 1A). After treatment with doramapimod, an inhibitor of p38 signaling, ZO-1 expression was significantly downregulated in the MEKK3-I441M-overexpressing HUVECs (Figures 2B,C and Supplementary Figures 1B,C).

Consistent with previous studies (20, 27), after *CCM2* was inactivated in HUVECs by lentivirus, Western blotting showed that ZO-1 expression was significantly decreased compared with that in the controls (Figure 2D and Supplementary Figure 1D), and immunofluorescence staining also showed that ZO-1 expression was obviously decreased after *CCM2* knockdown (Supplementary Figure 3A). In addition to ZO-1, Claudin-5, Occludin, and VE-cadherin also showed decreased expression levels after *CCM2* knockdown (Figure 2D and Supplementary Figure 1D). These findings suggest that *MAP3K3* mutation has different effects on ZO-1 expression compared with *CCM2* knockdown.

### *MAP3K3* mutation has distinct effects on TM expression compared with *CCM2* knockdown in HUVECs

Thrombomodulin (TM) is a 557-amino acid protein with a broad cell and tissue distribution consistent with its wide-ranging physiological roles. TM is expressed on the lumenal surface of vascular endothelial cells in both large vessels and capillaries, and its primary function is to mediate endothelial thromboresistance (28). Previous studies have shown that the TM levels are increased in human CCM lesions, as well as in the plasma of patients with CCMs. In mice, endothelial-specific genetic inactivation of *KRIT1* or *PDCD10*, which causes CCM formation, results in increased levels of vascular TM. Increased TM expression occurs because of the upregulation of transcription factors KLF2 and KLF4 consequent with the loss of *KRIT1* or *PDCD10*. Increased TM expression contributes to CCM hemorrhage (24).

**Figure 3:**
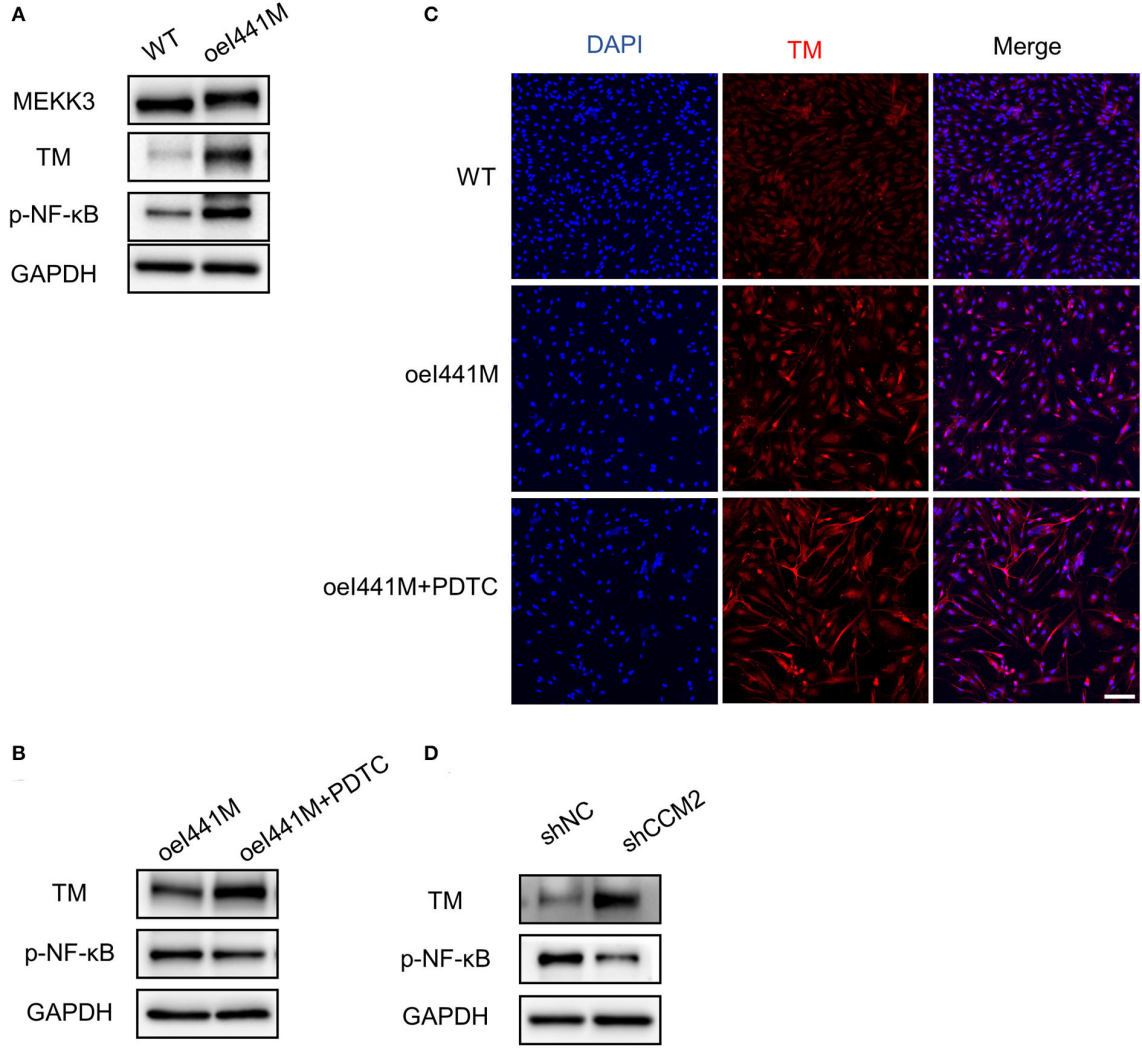
*MAP3K3* mutation has different effects on TM expression compared with *CCM2* knockdown in HUVECs. **(A–C)** HUVECs were infected by lentivirus with wild-type MEKK3 (WT) or MEKK3-I441M overexpression (oeI441M). Panel A shows that the expression of phospho-NF-κB (p-NF-κB) and TM was highly increased compared with that of WT. Panels B and C show that after treatment with pyrrolidinedithiocarbamate ammonium (PDTC), an NF-κB signaling inhibitor, TM expression was further upregulated. **(D)** Western blotting showed that compared with shNC, TM expression was highly increased in *CCM2*-knockdown HUVECs. HUVECs were infected with *CCM2*-knockdown lentivirus (sh*CCM2*) or a negative control (termed shNC). One representative experiment of 3 is shown. Scale bar, 200 μm.

To investigate the expression of TM in *MAP3K3* c.1323C>G mutation and *CCM2* knockdown endothelial cells, we infected HUVECs with lentivirus overexpressing MEKK3-I441M, WT, sh*CCM2*, and shNC, respectively. Previous studies have shown that KLF2 and KLF4 can increase TM expression and that activation of the NF-κB pathway can decrease TM expression (24, 29). In our study, compared with that in wild-type cells, after overexpression of MEKK3-I441M, the expression of KLF2, KLF4, and phospho-NF-κB was significantly increased, while Western blotting and immunofluorescence staining showed that TM expression was elevated in MEKK3-I441M-overexpressing HUVECs (Figures 3A,C and Supplementary Figures 2A,C). Interestingly, after treatment with pyrrolidinedithiocarbamate ammonium, an NF-κB signaling inhibitor, TM expression was further upregulated in MEKK3-I441M-overexpressing HUVECs (Figures 3B,C and Supplementary Figures 2B,C).

Consistent with a previous study (24), Western blotting in our study showed that compared with the control, TM expression was significantly increased and phospho-NF-κB was not activated in *CCM2*-knockdown HUVECs (Figure 3D and Supplementary Figure 2D). Immunofluorescence staining confirmed that TM expression was increased after *CCM2* knockdown (Supplementary Figure 3B). These findings suggest that *MAP3K3* mutation has distinct effects on TM expression compared with *CCM2* knockdown.

### Comparison of the expression of ZO-1 and TM in surgical CCM samples with *MAP3K3* and CCM gene mutations

**Figure 4:**
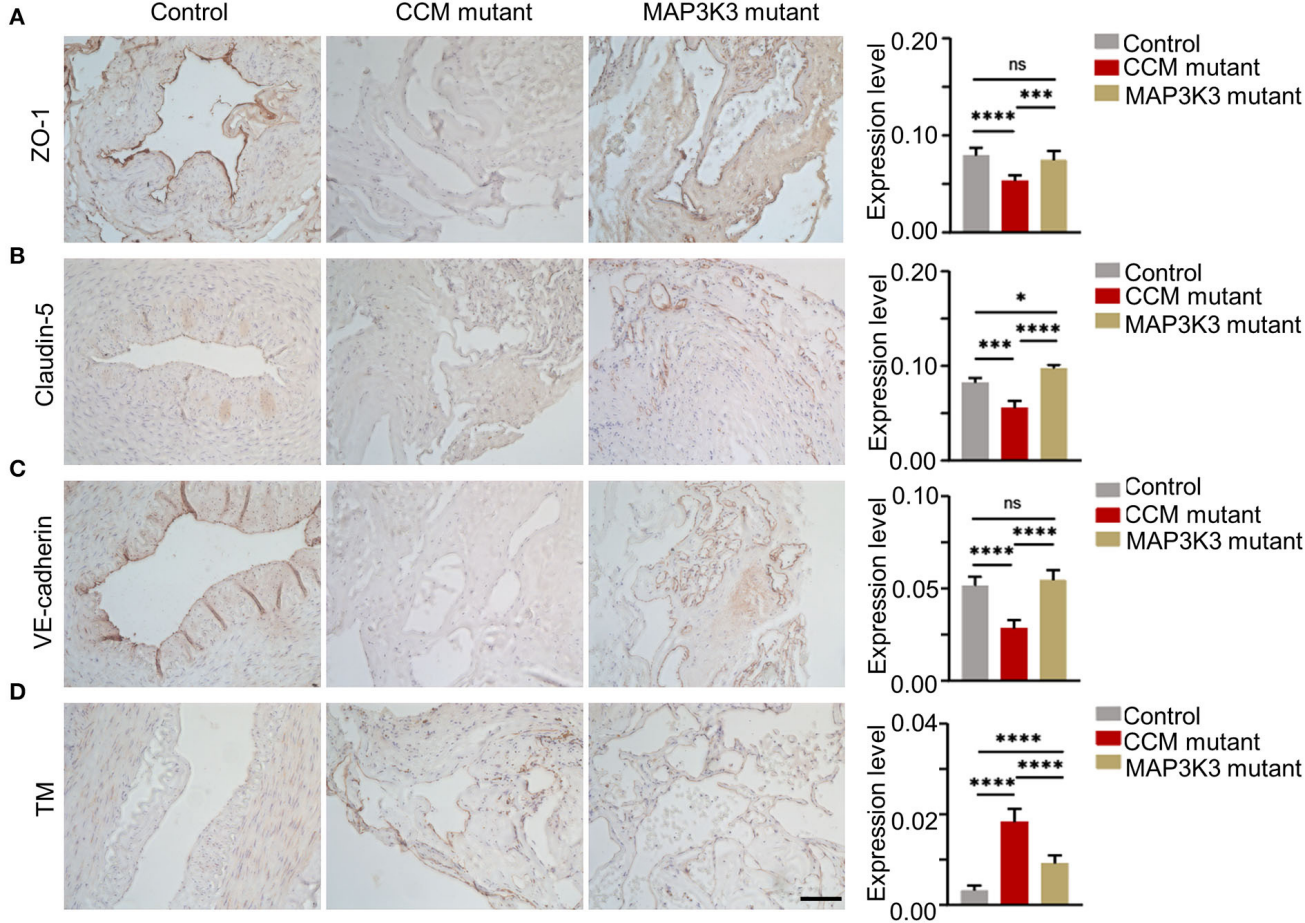
Comparison of ZO-1 and TM in surgical samples between *MAP3K3* and CCM gene mutations. **(A)** Immunohistochemical staining for ZO-1 in the CCM gene mutant, *MAP3K3* mutant and control samples. The histogram shows semiquantitative grading of the ZO-1 expression levels in the CCM gene mutant, *MAP3K3* mutant, and control samples. **(B)** Immunohistochemical staining for Claudin-5 in the CCM gene mutant, *MAP3K3* mutant, and control samples. The histogram shows semiquantitative grading of the Claudin-5 expression levels in the CCM mutant, *MAP3K3* mutant, and control samples. **(C)** Immunohistochemical staining for VE-cadherin expression in CCM gene mutant, *MAP3K3* mutant, and control samples. The histogram shows semiquantitative grading of the VE-cadherin expression levels in the CCM gene mutant, *MAP3K3* mutant, and control samples. **(D)** Immunohistochemical staining for TM in the CCM gene mutant, *MAP3K3*-mutant, and control samples. The histogram shows semiquantitative grading of the TM expression levels in the CCM gene mutant, *MAP3K3*-mutant, and control samples. Control indicates the superficial temporal artery (*n* = 3); *MAP3K3* mutant indicates CCM lesions with *MAP3K3* mutation (*n* = 3); CCM mutant indicates CCM lesions with CCM gene mutation (*n* = 3), including 1 *CCM1* and 2 *CCM2* mutant lesions. Scale bar, 200 μm. *p < 0.05; ***p < 0.001; ****p < 0.0001.

To validate the different effects of *MAP3K3* or CCM gene mutations on ZO-1 and TM expression, we performed immunohistochemical staining in surgical samples with *MAP3K3* mutation, CCM gene mutations, and normal arteries, including 3 *MAP3K3* mutant samples, 3 *CCM*-mutant samples, and 3 superficial temporal arteries as controls. Immunohistochemical staining showed that ZO-1 expression in the samples harboring CCM gene mutations was significantly lower than that in the control samples (*p* < 0.0001, *t*-test), whereas ZO-1 expression in the *MAP3K3-*mutant samples was not different from that in the control samples (*p* = 0.3810; *t*-test) (Figure 4A). The expression of Claudin-5 and VE-cadherin in the lesions with CCM gene mutations was significantly lower than that in the control samples (*p* < 0.05; *t*-test), while no difference was found in VE-cadherin expression in the *MAP3K3*-mutant samples compared with that in the control samples (*p* =0.2069; *t*-test) (Figures 4B,C). These findings suggest that the ZO-1 expression level is different between CCM-mutant lesions and *MAP3K3-*mutant lesions.

The TM expression level in both CCM gene and *MAP3K3* mutant samples was significantly higher than that in the control samples (*p* < 0.0001; *t*-test); however, the level of TM in the CCM gene mutant lesions was significantly higher than that in the *MAP3K3* mutant lesions (*p* < 0.0001; *t*-test) (Figure 4D). These findings suggest that the expression level of TM is different between CCM gene mutant lesions and *MAP3K3* mutant lesions.

Additionally, we performed immunohistochemical staining to detect the expression of angiogenic markers as Endoglin, VEGF, PCNA, HIF-alpha1, and Flk1 in CCM mutant lesions and normal superficial temporal arteries as control. Compared with control, the expression of Endoglin, VEGF, and PCNA was increased in CCM mutant lesions, and there was no significant difference in HIF-alpha1 and Flk1 expression between CCM mutant lesions and control (Supplementary Figure 4). Our results were consistent with previous studies (30–35).

## Discussion

In this study, we demonstrated that *MAP3K3* mutation presents distinct clinical characteristics compared with CCM gene mutations: *MAP3K3* mutation leads to less destruction of the brain–blood barrier, and less local anticoagulant molecule accumulation in the endothelium may explain its lower risk of hemorrhage events. Our results may imply that simplex CCMs have two distinct clinical subtypes.

Currently, molecular classifications are widely used in intracranial tumors, particularly in gliomas. A study reported that the molecular classification of glioblastoma involving IDH1, PDGFRA, EGFR, and NF1 substantially benefits the prediction of prognosis and response to therapy in glioblastoma patients (36). Vascular anomalies can be caused by inherited or somatic genetic mutations (13, 37, 38). The identification of inherited and somatic mutations in vascular anomalies has led to the evaluation of tailored strategies with preexisting cancer drugs that interfere with these signaling pathways (39). However, the molecular classification of simplex vascular diseases has not yet been well established. In this study, we indicated that simplex CCMs might comprise two clinical subtypes relevant to CCM genes and *MAP3K3* somatic mutations. The two subclasses of simplex CCMs had different risks of hemorrhage events: CCM gene mutant lesions were susceptible to frequent overt hemorrhage, whereas *MAP3K3* mutant lesions rarely led to overt hemorrhage and remained stable. Furthermore, somatic mutations of the two genotypes demonstrated different effects on the anti-coagulation and TJs in the endothelium, at least partially explaining the underlying mechanism of the specific clinical manifestations. Our findings may contribute to predicting the prognosis and treatment choices in patients with simplex CCMs.

Our study had some limitations. The genotypes of simplex CCMs are difficult to obtain in patients under long-term observation: we have difficulties to have surgical samples from long-term follow-up patients. Therefore, we are not able to investigate the lesion evolution with confirmed genotypes in a long-term follow-up in a large cohort, and larger cohort studies are needed to further strengthen the results of this study. The expression levels of TJs proteins and TM consequent to CCM gene mutation and *MAP3K3* mutation were limited to *in vitro* experiments, and the findings required future studies in animal models.

## Conclusion

Compared with CCM gene mutations, simplex CCMs with *MAP3K3* mutation occasionally present with overt hemorrhage, which is associated with the biological function of *MAP3K3* mutation in the endothelium. Future studies in animal models and larger cohort CCMs are needed to further strengthen the results of this study.

## Data availability statement

The original contributions presented in the study are included in the article/Supplementary material, further inquiries can be directed to the corresponding author/s.

## Ethics statement

The studies involving human participants were reviewed and approved by Institutional Review Board of Tiantan Hospital. Written informed consent to participate in this study was provided by the participants' legal guardian/next of kin. The animal study was reviewed and approved by Institutional Review Board of Tiantan Hospital. Written informed consent was obtained from the individual(s) for the publication of any potentially identifiable images or data included in this article.

## Author contributions

RH and JW designed the study, conducted experiments, analyzed and interpreted the data, and drafted the manuscript for intellectual content. Y-FS, J-CW, HL, and Y-MJ collected and interpreted the data and revised the manuscript for intellectual content. H-YX, J-ZZhan, S-ZZ, and Q-HH collected the data and revised the manuscript for intellectual content. SW and J-ZZhao designed the study and revised the manuscript for intellectual content. YC provided overall oversight of the research. All authors contributed to the article and approved the submitted version.

## Funding

This study was supported by the National Natural Science Foundation of China (Grant No. 82171267) and the National Key Research and Development Program of China (Grant No. 2021YFC2501102).

## Conflict of interest

The authors declare that the research was conducted in the absence of any commercial or financial relationships that could be construed as a potential conflict of interest.

## Publisher's note

All claims expressed in this article are solely those of the authors and do not necessarily represent those of their affiliate organizations, or those of the publisher, the editors and the reviewers. Any product that may be evaluated in this article, or claim that may be made by its manufacturer, is not guaranteed or endorsed by the publisher.

## Abbreviations

CCMs: cerebral cavernous malformations
HUVECs: human umbilical vein endothelial cells
MRI: magnetic resonance imaging
TM: thrombomodulin
TJs: tight junctions.
