# Supplementary material for: Simplex cerebral cavernous malformations with MAP3K3 mutation have distinct clinical characteristics
Source: Front Neurol. 2022 Aug 26;13:946324. doi: 10.3389/fneur.2022.946324 (PMC9458974; doi:10.3389/fneur.2022.946324)
Supplement: Supplementary file 1 [file Data_Sheet_1.PDF]

Supplementary Material

Simplex cerebral cavernous malformations with MAP3K3 mutation have distinct clinical characteristics

Supplementary Figures

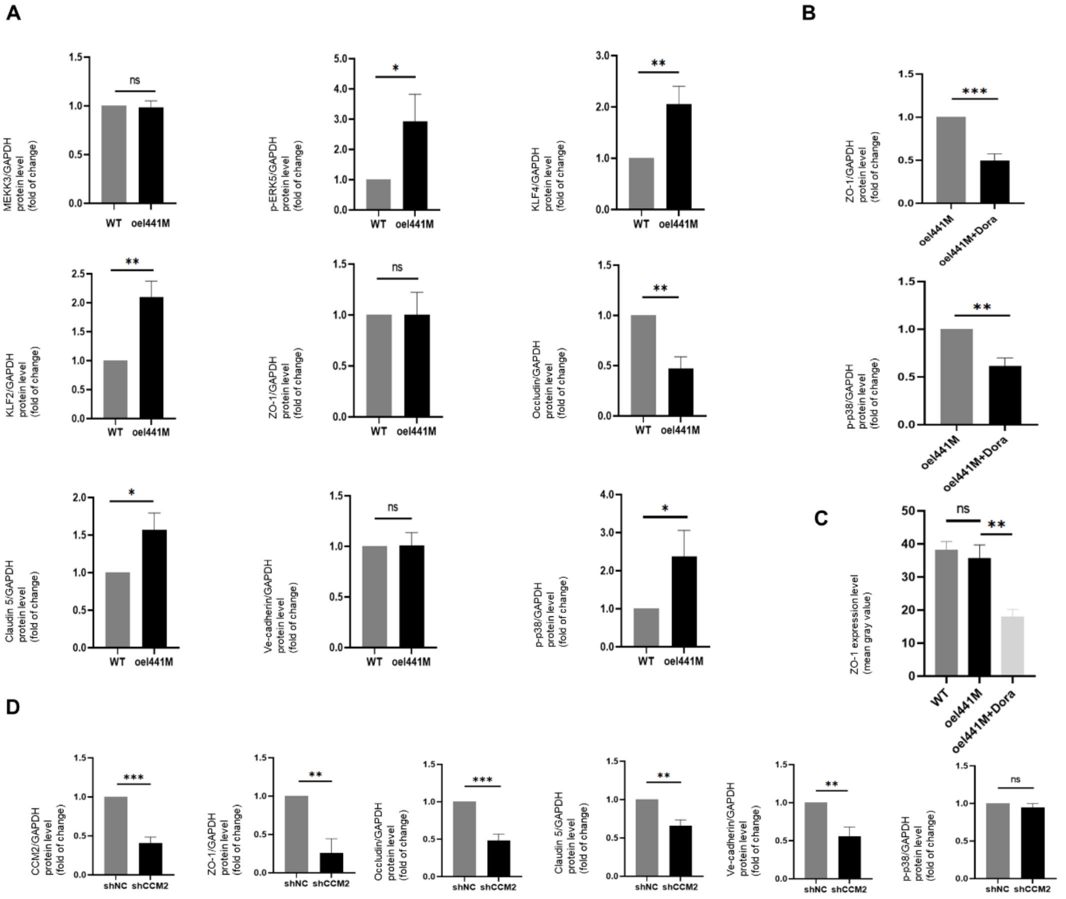

Supplementary Figure 1. Quantitation histogram of relative protein expression between different group. A. Quantitation histogram of relative expression of MEKK3, p-ERK5, KLF4, KLF2, ZO-1, Occuldin, Claudin 5, Ve-cadhrin, p-p38 by Western blotting between WT and oeI441M group. B. Quantitation histogram of relative expression level of ZO-1 and p-p38 by Western blotting between oeI441M and oeI441+ Dora group. C. Quantitation histogram of relative expression protein of ZO-1 by immunofluorescence staining in WT, oeI441M, oeI441M+Dora group. D. Quantitation histogram of relative expression protein of CCM2, ZO-1, Occuldin, Claudin 5, Ve-cadhrin, p-p38 by Western blotting between shNC and shCCM2 group. All values were expressed as the mean±SD. *p<0.05; **p<0.01; ns, no significance.

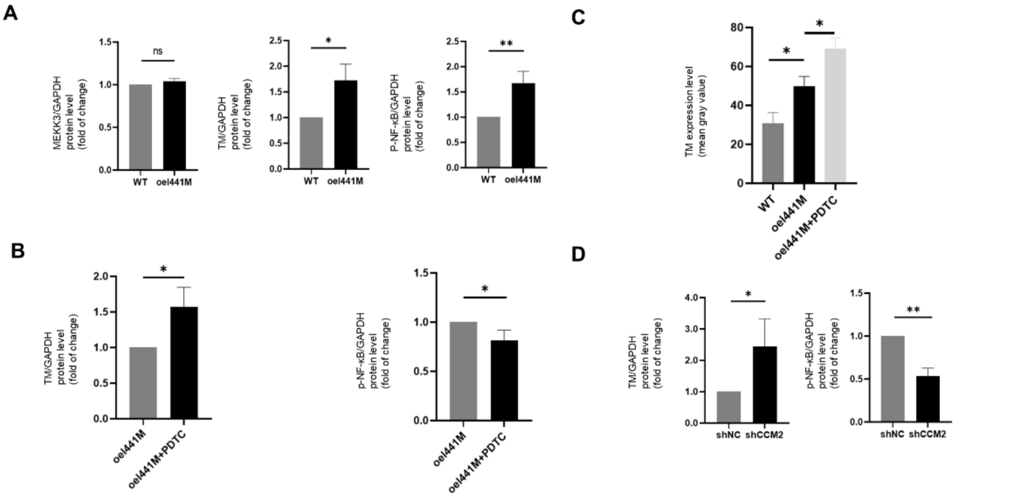

Supplementary Figure 2. Quantitation histogram of relative protein expression between different group. A. Quantitation histogram of relative expression of MEKK3, TM, and p-NF-κB, detected by Western blotting between WT and oeI441M group. B. Quantitation histogram of relative expression level of TM and p-NF-κB detected by Western blotting between oeI441M and oeI441+ PDTC group. C. Quantitation histogram of relative expression protein of TM detected by immunofluorescence staining in WT, oeI441M, oeI441M+PDTC group. D. Quantitation histogram of relative expression protein of TM and p-NF-κB detected by Western blotting between shNC and shCCM2 group. All values were expressed as the mean±SD. *p<0.05; **p<0.01; ns, no significance.

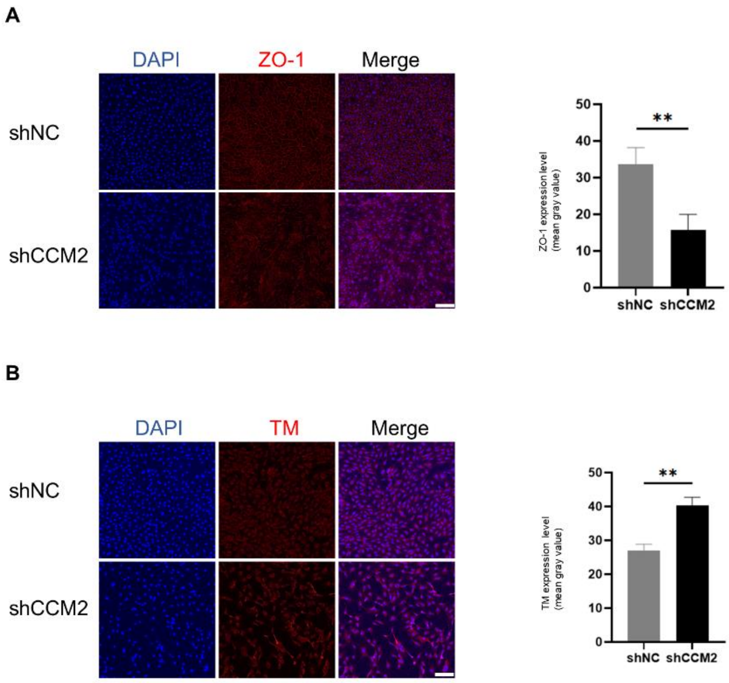

Supplementary Figure 3. Expression level of ZO-1 and TM between shNC and shCCM2 group. A. Expression level of ZO-1 detected by immunofluorescence staining and the quantitation histogram between shNC and shCCM2 group. B. Expression level of TM detected by immunofluorescence staining and the quantitation histogram between shNC and shCCM2 group. Scale bar, 200 μm. All values were expressed as the mean±SD. **$p<0.01$.

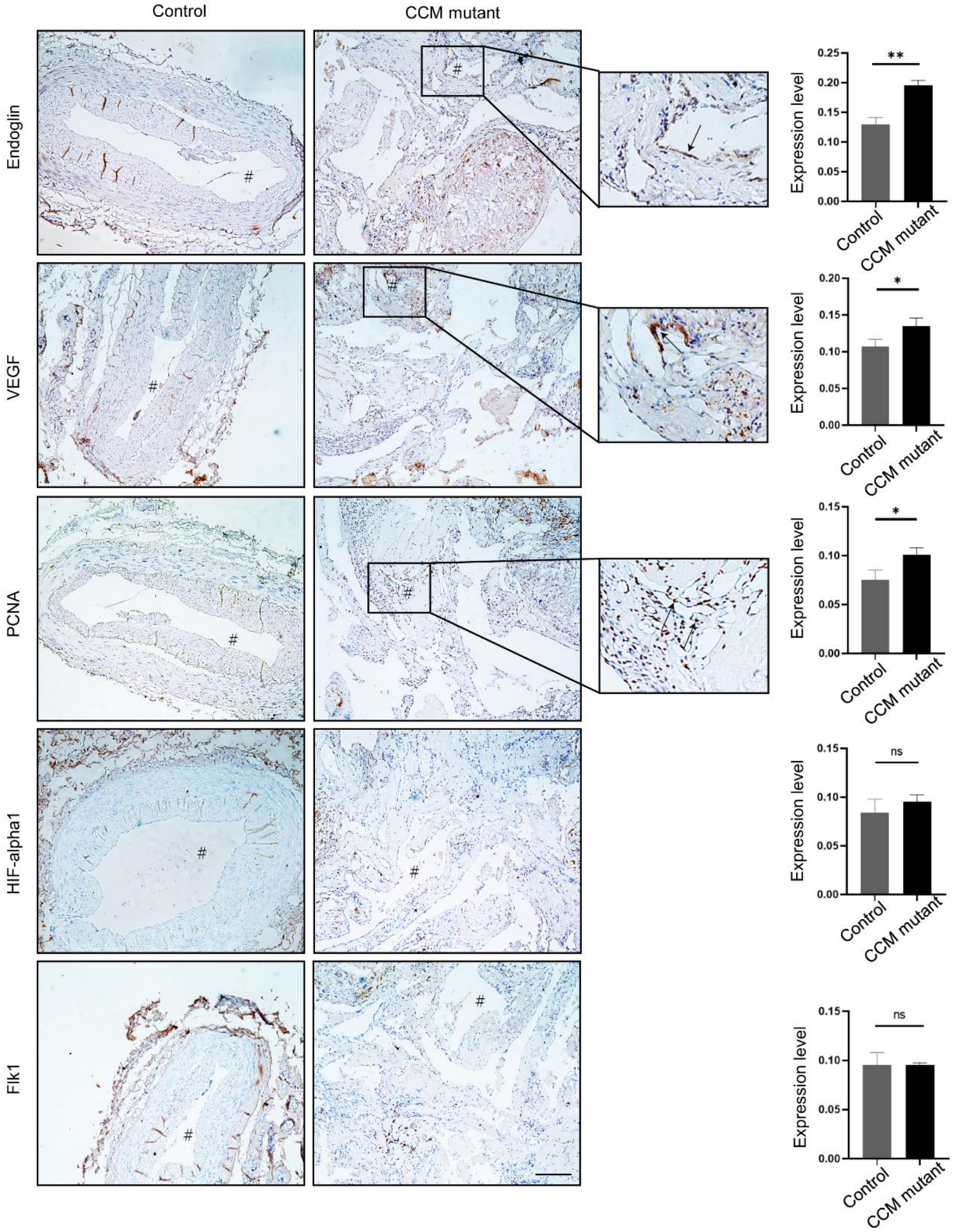

Supplementary Figure 4. Endoglin, VEGF, PCNA, HIF-alpha1, and Flk1 expression and quantitation histogram between CCM mutant lesions and control superficial temporal arteries. Control indicates the superficial temporal artery (n=3); CCM mutant indicates CCM lesions with CCM gene mutation (n=3), including 1 CCM1 and 2 *CCM2* mutant lesions. The black arrow indicated positive area. Scale bar, 200 μm. All values were expressed as the mean±SD. #vessel lumen; *p<0.05; **p<0.01; ns, no significance.

Supplementary Tables

Supplementary Table 1. Detailed gene mutation information for each sample of 50 patients.

ID	CCM gene or MAP3K3 mutation	Mutant frequency by WES	Mutant frequency by ddPCR	PIK3CA mutation	Mutant frequency by WES	Mutant frequency by ddPCR
p1	MAP3K3	0	0.09	NO	0	NA
p2	MAP3K3	11.00	15.37	NO	0	NA
p3	MAP3K3	6.00	7.5	YES	8.36	NA
p4	MAP3K3	1.00	0.93	YES	3.77	NA
p5	MAP3K3	15.00	16.02	YES	20.55	NA
p6	MAP3K3	1.00	1.07	NO	0	NA
p7	MAP3K3	1.00	1.59	NO	0	NA
p8	MAP3K3	0.60	1.09	NO	0	NA
p9	MAP3K3	13.00	9.18	NO	0	NA
p10	CCM1	4.00	NA	NO	0	NA
p11	CCM2	4.00	NA	NO	0	NA
p12	CCM1	10.26	NA	YES	8.24	NA
p13	CCM2	11.35	NA	NO	0	NA
p14	CCM1	9.68	NA	YES	15.29	NA
p15	CCM2	5.00	NA	NO	0	NA
p16	CCM2	1.00	NA	YES	4.36	NA
p17	MAP3K3	NA	1.44	NA	NA	NA
p18	MAP3K3	NA	1.91	NA	NA	NA
p19	MAP3K3	NA	4.21	NA	NA	NA
p20	MAP3K3	NA	0.75	NA	NA	NA
p21	MAP3K3	NA	5.83	NA	NA	NA
p22	MAP3K3	NA	1.16	NA	NA	NA

p23	MAP3K3	NA	8.03	NA	NA	NA
p24	MAP3K3	NA	3.20	NA	NA	NA
p25	MAP3K3	NA	8.07	NA	NA	NA
p26	MAP3K3	NA	2.00	NA	NA	NA
p27	MAP3K3	NA	0.67	NA	NA	NA
p28	MAP3K3	NA	5.46	NA	NA	NA
p29	MAP3K3	NA	9.83	NA	NA	NA
p30	MAP3K3	NA	4.44	NA	NA	NA
p31	MAP3K3	NA	5.08	NA	NA	NA
p32	MAP3K3	NA	1.97	NA	NA	NA
p33	MAP3K3	NA	0.08	NA	NA	NA
p34	MAP3K3	NA	13.47	NA	NA	NA
p35	MAP3K3	NA	0.22	NA	NA	NA
p36	MAP3K3	NA	9.89	NA	NA	NA
p37	MAP3K3	NA	2.60	NA	NA	NA
p38	MAP3K3	NA	0.03	NA	NA	NA
p39	MAP3K3	NA	4.57	NA	NA	NA
p40	MAP3K3	NA	5.08	NA	NA	NA
p41	MAP3K3	NA	9.97	NA	NA	NA
p42	MAP3K3	6.92	9.82	YES	9.01	NA
p43	MAP3K3	2.96	2.26	YES	4.41	1.99
p44	MAP3K3	4.93	NA	YES	4.41	NA
p45	CCM2	1.85	NA	YES	3.21	NA
p46	CCM1	5.08	NA	NO	0	NA
p47	CCM1	3.09	NA	YES	2.47	0.79
p48	CCM1	2.04	NA	YES	10.67	6.05

p49	MAP3K3	0	5.67	YES	8.18	6.2
p50	CCM1	4.22	NA	YES	6.25	NA

Abbreviations: WES, whole-exome sequencing; ddPCR, droplet digital polymerase chain reaction; NA, not available.

Supplementary Table 2. Clinicopathological features between brainstem and supratentorial CCM lesions in different gene mutation group.

Variables	Overall	CCM gene mutation		p values	MAP3K3 mutation		p values
	(n=46)	Supratentorial(n=8)	Brainstem (n=1)		Supratentorial(n=29)	Brainstem (n=8)	
Age-mean-yr	32.5±16.2	32.8±14.2	56	NA	32.7±16.7	29.0±16.6	0.587†
Female-no.(%)	16(34.8)	3(37.5)	1(100)	0.444‡	9(31.0)	3(37.5)	1.000‡
Hemorrhage events-no.(%)	17(37.0)	7(87.5)	1(100)	1.000‡	6(20.7)	3(37.5)	0.373‡
Size-mean-mm	23.0±8.7	24.0±7.9	23	NA	23.2±9.6	21.1±7.3	0.566†
Main complaint-no.(%)				0.556‡			<0.001‡*
Epilepsy	24(52.1)	4(50.0)	0		20(69.0)	0	
FND	14(30.4)	2(25.0)	1(100)		3(10.3)	8(100)	
Headache	6(13.0)	1(12.5)	0		5(17.2)	0	
Others	2(4.3)	1(12.5)	0		1(3.4)	0	

Abbreviations: FND, focal neurological deficit; NA, not available; MAP3K3 mutation, MAP3K3 (c.1323C>G [p.Ile441Met]) somatic mutation; CCM gene mutation, CCM1/KRIT1 or CCM2/MGC4607 somatic mutation; NA, not available; †t-test; ‡Fisher's exact test; *p<0.05.
